# Metalenses Based on Symmetric Slab Waveguide and c-TiO_2_: Efficient Polarization-Insensitive Focusing at Visible Wavelengths

**DOI:** 10.3390/nano8090699

**Published:** 2018-09-07

**Authors:** Yaoyao Liang, Zhongchao Wei, Jianping Guo, Faqiang Wang, Hongyun Meng, Hongzhan Liu

**Affiliations:** 1 Guangzhou Key Laboratory for Special Fiber Photonic Devices, South China Normal University, Guangzhou 510006, China; lyy777@m.scnu.edu.cn (Y.L.); guojp@scnu.edu.cn (J.G.); fqwang@scnu.edu.cn (F.W.); hymeng@scnu.edu.cn (H.M.); lhzscnu@163.com (H.L.); 2 Guangdong Provincial Key Laboratory of Nanophotonic Functional Materials and Devices, School of Information and Optoelectronic Science and Engineering, South China Normal University, Guangzhou 510006, China

**Keywords:** metasurface, metalens, phase modulation, integrated devices, crystalline titanium dioxide

## Abstract

A key goal of metalens research is to achieve wavefront shaping of light using optical elements with thicknesses on the order of the wavelength. Here we demonstrate ultrathin highly efficient crystalline titanium dioxide metalenses at blue, green, and red wavelengths (*λ*_0_ = 453 nm, 532 nm, and 633 nm, respectively) based on symmetric slab waveguide theory. These metalenses are less than 488 nm-thick and capable of focusing incident light into very symmetric diffraction-limited spots with strehl ratio and efficiency as high as 0.96 and 83%, respectively. Further quantitative characterizations about metalenses’ peak focusing intensities and focal spot sizes show good agreement with theoretical calculation. Besides, the metalenses suffer only about 10% chromatic deviation from the ideal spots in visible spectrum. In contrast with Pancharatnam–Berry phase mechanism, which limit their incident light at circular polarization, the proposed method enables metalenses polarization-insensitive to incident light.

## 1. Introduction

Conventional objectives generally consist of precision-engineered compound lenses which make them bulky and costly, limiting their applications and hindering their integration into compact and cost-effective systems. In recent years, metasurfaces, which refer to a two-dimensional metamaterial system, have emerged as one of the leading platforms for the development of miniaturized optical components due to their conspicuous features that flexibly tailor optical properties in terms of the polarization, intensity, and phase [1]. Since the phase is accurately controlled by subwavelength spaced structures with thicknesses at the wavelength scale or below, many compact optical devices based on metasurfaces such as flat lenses (metalens) [2,3,4], axicons [5,6], waveplates [7,8], and vortex plates [9] have been reported. Besides, significant progress has been made recently regarding metasurfaces in their component materials from the plasmonic noble metals [10,11,12,13,14,15,16] to silicon [17,18,19] and to other new all dielectric stuffs like silicon nitride (Si_3_N_4_) [20], gallium nitride (GaN) [21,22], and amorphous titanium dioxide (a-TiO_2_) [23,24] and so on, which further open avenues for the development of high-efficiency devices in the visible spectrum. In fact, up to now, most of the advanced metalenses with large numerical apertures (NA) [23,25,26,27] were usually demonstrated based on the Pancharatnam–Berry (P-B) [28] phase mechanism because of the clear and convenient geometry phase of antenna as a function of position *φ* = 2*θ*, where *θ* is the rotational angle of the antenna, one can easily relate the required phase *φ* and *θ* to build the metalenses in the code programming process. However, this phase mechanism relies deeply on the cross-polarization of the incident circular polarized light, the copolarized component of the incident field does not participate in the focusing process, which would theoretically result in the extra loss of total incident power in addition to the common losses like absorptions and reflections. Fortunately, metalenses [29] based on waveguide phase can solve this problem by fully utilizing the power of incident light (not including common losses like absorptions, reflections, and so on) to focus, thus it can enable the metalenses to be highly efficient at initial design. Whereas, these waveguide-type metalenses, especially two dimension (2D) large-NA metalenses, are difficult to build because there is not an as convenient relationship between the required phase and antenna as P-B mechanism. As a result, many previous metalenses with this type were just simply designed by the 1D metasurfaces with several antennas [30,31,32,33,34] in a period or 2D metasurfaces with dozens of antennas in whole [20,35,36], however, these simple and insufficient arrays cannot produce as good wavefronts as possible to ensure the high performance of metalenses that are comparable or even superior to conventional lenses (see Figure S1). In this case, their focusing effects were hard to quantitatively analyze and usually were qualitatively demonstrated. 

In this paper, we have designed and demonstrated highly efficient metalenses operating in the visible spectrum based on a promising material-crystalline titanium dioxide (c-TiO_2_) and a new phase modulation mechanism-symmetric slab waveguide (SSW) theory. The metalenses are ultrathin with 488 nm thickness and have the capacity of focusing the incident light into very symmetric focal spots with Strehl ratio and focusing efficiency as high as 0.96 and 83%, respectively. Besides, comparing previous evaluations on focusing performance, most were qualitative discussed, we characterize the metalenses in a more detailed ways by quantitatively researching the metalenses’ peak focusing intensities (PFI) and focal spot sizes. Simulation results agreeing well with theoretical calculations prove the high performance of proposed metalenses based on SSW theory. Furthermore, in contrast with P-B metalenses, which limit their incident light at circular polarization state, the proposed method enable metalenses polarization-insensitive to incident light, no matter the linear polarization from any state (0–360°) or circular polarization with different chirality.

## 2. Methods

**Figure 1:**
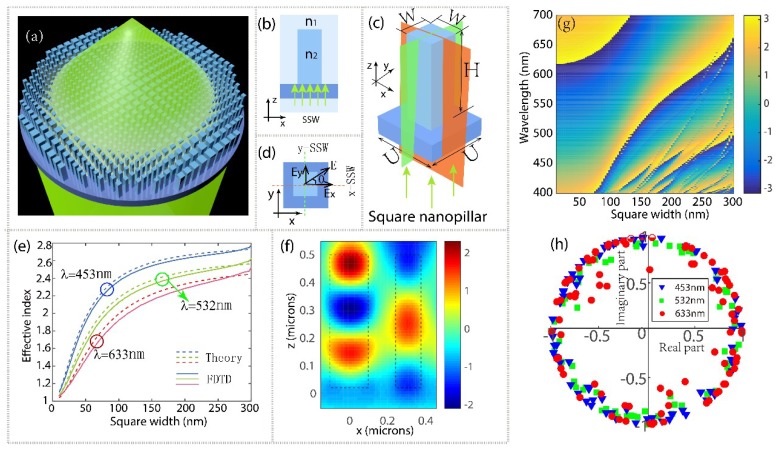
(**a**) The schematic of a metalens focuses incident light into a spot in transmission mode. (**b**) Schematic diagram of a symmetric slab waveguide on a glass substrate, the waveguide consists of a guiding layer of refractive index *n*_2_, surrounded by media of refractive index *n*_1_. (**c**) Schematic diagram of nanoantenna of proposed metalenses, a square crystalline titanium dioxide nanopillar on a glass substrate. The orange and green planes represent the SSW models in the x and y direction, respectively. (**d**) Top view of the nanopillar in (c). (**e**) Effective index of square nanopillar as a function of its width *W* for different wavelengths of 453, 532, and 633 nm calculated by FDTD solutions (soild lines) and SSW theory (dotted lines), respectively. (**f**) Electric near field (real [*Ex*]) distribution in xz-plane. Left and right pillars have widths of 250 nm and 150 nm, respectively. Boundaries of the nanopillars are highlighted by vertical dotted lines. Simulations are performed under plane-wave excitation and parallel polarization (electric field along the x) at *λ*_0_ = 633 nm. Perfectly matched layer is used for z and periodic boundary conditions are used for x and y, respectively. (**g**) Phase map as a function of square width of nanopillar across the visible spectrum. (**h**) Complex transmission coefficients for three design wavelengths. Each point represents the amplitude and phase of the transmission of a square nanopillar with width *W*. Here all metalenses are *H* = 488 nm tall and the sampling spaces U are optimized to 180 nm for blue metalens, 300 nm for green and red metalenses, respectively.

The building blocks of metalens are c-TiO_2_ nanopillars on a glass substrate (Figure 1a). The metalens focus incident light into a spot in transmission mode. To accomplish this, each nanopillar at position ($x_{i},y_{i}$) must impart the required phase given by:

(1) $$\varphi (x_{i},y_{i},\lambda_{0})=2l\pi +\frac{2\pi }{\lambda_{0}}(f_{0}-\sqrt{{f_{0}}^{2}+{x_{i}}^{2}+{y_{i}}^{2}})$$

where *l* is an arbitrary integer number, (*x_i_*, *y_i_*) are the in-plane coordinates, *λ*_0_ and *f*_0_ are the design wavelength and focal length, respectively.

To gain a better insight into the phase realization mechanism, here we introduce a simple but efficient model with symmetric slab waveguide (SSW) effect. Figure 1b shows a typical example of a SSW on the glass substrate with the propagation direction along the +z. It consists of a thin dielectric layer (called the guiding layer, or simply the core) with a refractive index *n*_2_, sandwiched between two semi-infinite bounding media (clad) of refractive index *n*_1_. By changing the core width, the effective index of SSW will vary accordingly. Inspired by this slab model, we proposed square nanopillar (Figure 1c) as metalens’ antenna, which can work as two same SSW models (green and orange planes) perpendicular in the x and y direction simultaneously. As shown in Figure 1d, the incident light with polarization angle of *θ* can be decomposed into x-component and y-component with their electric field of *E_x_* and *E_y_*, respectively, that will be independently responded by according slab waveguides, which guarantees polarization-insensitive operation of the square nanopillars. Besides, using square cross section of the nanopillars maximizes the filling factor range, from zero (no nanopillar) to 1 (width equals to center-to-center distance), which is necessary to increase the phase coverage [24]. 

Here we calculate the effective index of square nanopillar as a function of its width for different wavelengths of 453, 532, and 633 nm by FDTD solutions and SSW theory, respectively. For SSW theory, we derive the relationship between *β* and *w* as the following formula [37],

(2) $$2\arctan(\sqrt{\frac{4m}{h^{2}}-1})/h=w$$

in which *β* is the propagation constant of the slab waveguide, *w* is the square pillar width,

(3) $$m=\pi^{2}({n_{2}}^{2}-{n_{1}}^{2})/\lambda^{2}$$

(4) $$h={\left[{\left(\frac{2\pi n_{2}}{\lambda }\right)}^{2}-\beta^{2}\right]}^{1/2}$$

As shown in Figure 1e that by adjusting the square nanopillar width, the effective index of its fundamental mode can vary anywhere from *n_eff_* ≈ 1 (when the light is mostly in air) to *n_eff_* ≈ *n*_TiO_2__ (when the light is mostly in TiO_2_). It is apparent that the two methods fit well. If we have two pillars side-by-side, with different widths and negligible optical coupling between the nanopillars, then light traveling through the different pillars will accumulate as a phase shift ∆*φ* proportional to their length *H* [38],

(5) $$\Delta \varphi =\beta H=\frac{2\pi }{\lambda }\Delta n_{eff}H$$

where ∆*n_eff_* is the effective index difference between the two pillars. Figure 1f depicts the emergence of the phase difference between two waveguides of different widths. It is notable that due to high mode confinement and large index difference together with the short propagation distance, the TiO_2_ waveguide can confine light in a subwavelength region, which enables the antennas (nanopillars) to be packed very densely, with subwavelength separation and minimal coupling.

To ensure high efficiency, parameters such as the unit cell size (or sampling space) *U* is optimized at the design wavelength (see Figure S2). In this design, the phase accumulation is realized by means of the waveguiding effect; the height of the nanopillars should be tall enough to provide 2π phase coverage through a range of square widths, here we adapt the height from a past paper [39]. Figure 1g show the phase map as a function of square width of nanopillar across the visible spectrum. Obviously, full phase control can be realized across most visible wavelengths. To build the metalenses, we discretized its required phase *φ* (*x_i_, y_i_*) assuming square lattice unit cells of dimensions *U* × *U*. At each position (*x_i_*, *y_i_*) an appropriate square width *w*, which minimize |*T_a_e^iφ^*^(*xi,yi*)^ − *T(w)e^iφ^*^(*w*)^| was chosen, where *T_a_* is the transmission averaged over all the square widths. Figure 1h shows the complex transmission coefficients (*T*(*w*)*e^iφ^*^(*w*)^)at the three design wavelengths for a range of square widths required to give 2π phase coverage. Each point in the complex plane represents the amplitude and phase of the transmission of a nanopillar with square width *w*, for a given unit cell size and nanopillar height at the corresponding design wavelength. High transmission and close to 2π phase coverage is evident for all three design wavelengths. 

## 3. Results 

**Figure 2:**
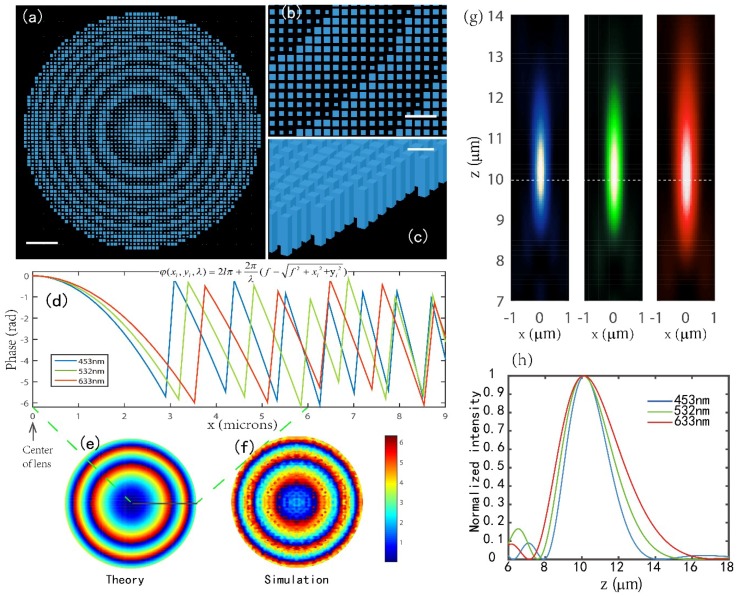
(**a**) Top-view image of the center portion of a metalens with NA = 0.75 and *f*_0_ = 10 μm built in FDTD solution environment. Scale bar: 2.4 μm. (**b**) Top-view image of a portion of the metalens at a higher magnification than that in (a), displaying each individual nanopillar. Scale bar: 900 nm. (**c**) Side view image of the edge of the metalens in (a), showing the vertical profile of the nanopillars. Scale bar: 500 nm. (**d**) The required phase profile to realize metalenses (NA = 0.51) at the three design wavelengths (*λ*_0_ = 453, 532, and 633 nm). (**e**,**f**) The calculated ideal phase distribution at the transmitted facet (**e**) and the simulated phase distribution at the plane 12 nm above the transmitted facet (**f**) of metalens design for *λ*_0_ = 633 nm. (**g**,**h**) Simulated intensity profiles (x-z plane) in the focal region (**g**) and along z axis (**h**) for three metalenses respectively, showing the simulated focal lengths are almost the design value of 10 μm.

Figure 2a–c show the metalens built in FDTD solution environment with NA = 0.75 and $f_{0}$ = 10 μm. Due to the large size of the proposed metalenses and our limited computational resources, we analyzed the performance of the metalenses with similar $f_{0}$ = 10 μm but smaller NA. Three distinct metalenses with NA = 0.51 were demonstrated with respective design wavelengths of 453, 532, and 633 nm. Figure 2d shows the required phase profile to realize metalenses at the three design wavelengths. Figure 2e is the ideal phase distribution at the transmitted facet of metalens design for *λ*_0_ = 633 nm, Figure 2f is the simulated result of phase distribution at the plane which is 12 nm above the transmitted facet of metalens of 633 nm. We can note that the designed metalens can work out very symmetric phase distribution as expected, which directly ensures the high symmetry of the focusing spots for designed metalenses. Figure 2g,h show intensity distributions in the focal region (x-z plane) and along z-axis for the three metalenses, respectively. It is clear that the three metalenses function well, as expected, and that the simulated focal spots arrive accurately at the design position of $f_{0}$ = 10 μm.

**Figure 3:**
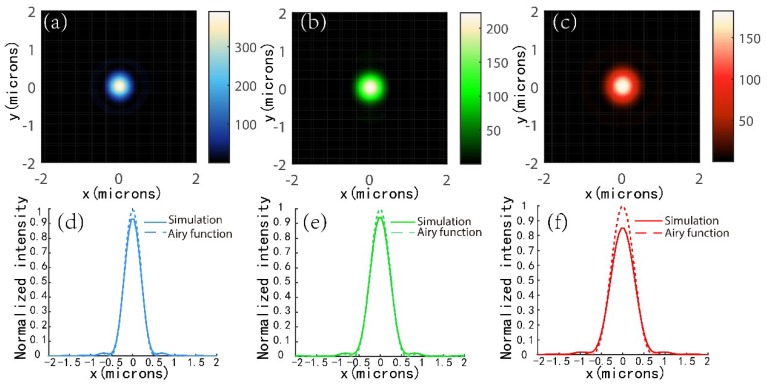
(**a**–**c**) simulated focal spots of the metalenses at their design wavelengths of (**a**) 453, (**b**) 532, and (**c**) 633 nm, respectively. (**d**–**f**) Corresponding horizontal cuts of focal spots shown in (**a**–**c**) with full width at half-maxima of 470, 550, and 650 nm, respectively. An ideal Airy function is overlaid onto each horizontal cut.

Figure 3a–c show highly symmetric focal spots obtained for three metalenses. Their vertical cuts of the focal spots are also shown in Figure 3d–f with full-width at half-maximum (FWHM) of 470, 550, and 650 nm, respectively. To characterize the focusing performance, the simulated intensities of the vertical cuts are normalized to those of ideal Airy functions with the same area under the curve. Airy functions with a maximum intensity of *I*_0_*= PT_λ_S/λ*_0_^2^*f*_0_^2^ and diffraction-limited full width at half-maximum = *λ*_0_/2*NA* are plotted over the horizontal cuts, here *P* is the total power incident on the metalens and can be expressed as $P=\int_{S}EdS$, *T_λ_* is the metalens’ transmission at design wavelength, and *S* is the area of metalens. Strehl ratios (the ratio of simulated PFI to that of according ideal value) of 0.96, 0.94, and 0.85 are achieved at wavelengths of 453, 532, and 633 nm, respectively. In addition, simulated focusing efficiencies as high as 83.4%, 73.5%, and 78% are obtained for the three metalenses, respectively. The efficiency is calculated as the ratio of the optical power in the focal spot region (circle of radius 2 × FWHM spanning the center of the focal spot) to that of the incident beam. The incident beam was defined as the optical power passing through a circular aperture with the same diameter as the metalenses.

**Figure 4:**
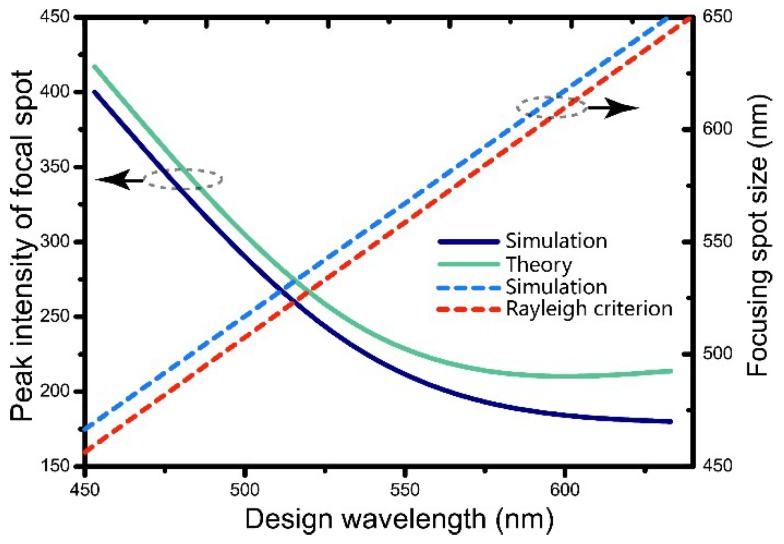
Solid lines represent the simulated (dark blue line) and theoretical (green line) PFIs of metalenses operating at different design wavelengths. Dotted lines show the simulated focal spot sizes of the proposed metalenses (red dotted line) and the according diffraction-limited sizes (blue dotted line) at different wavelengths. All the lenses have the same NA of 0.51 and *f*_0_ of 10 μm. (For further information refer to Figure S3).

We also quantitatively show the comparison in Figure 4 of the simulation results and the theoretical calculation of the PFIs and sizes of focal spots for different design wavelengths ranging from 450 nm to 640 nm. It is notable that the PFIs obtained by the two methods consistently decrease, especially for smaller wavelengths, with increasing design wavelength. Note that the simulated focal spot sizes are only about 20 nm lager than the diffraction-limited values (Rayleigh criterion) at different design wavelengths, which proved the high focusing performance of the metalenses based on the proposed slab waveguide model.

## 4. Discussions

### 4.1. Characterization of Aberration

**Figure 5:**
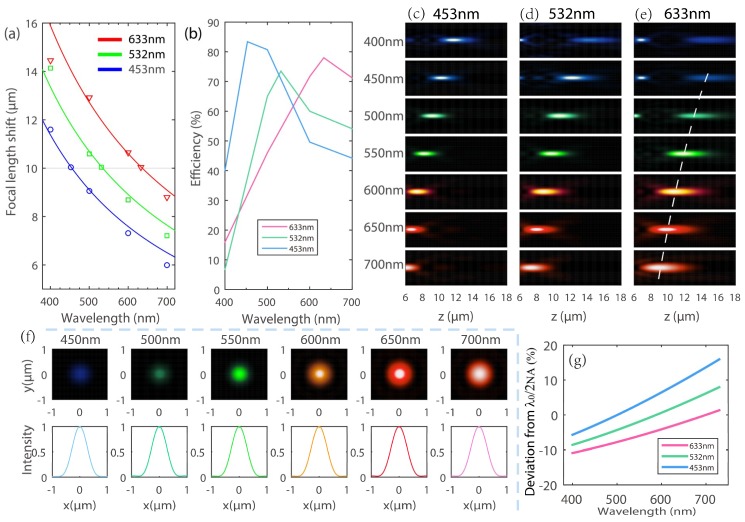
(**a**) Simulated focal length shifts (symbols) of metalenses designed for *λ*_0_ = 453, 532, and 633 nm compared to diffractive lens (lines) according to Equation (6), respectively. (**b**) The wavelength dependence of focusing efficiency for the three metalenses at visible spectrum. All the metalenses have the same NA of 0.51 and focal length of 10 μm. (**c**–**e**) Intensity distributions in line scale (in false colors corresponding to their respective wavelengths) in the x-z plane of the three metalenses at different wavelengths ranging from 400 nm to 700 nm, respectively. The wavelengths of incidence are denoted on each picture. The direction of incidence is towards the positive z-axis. (**f**) Normalized intensity profiles along the white dashed lines of (**e**) for metalenses, respectively, designed for *λ*_0_ = 633 nm. The white dashed lines pass through the center of focal spots. (**g**) The chromatic deviations from the ideal spots for three metalenses in visible spectrum.

The metalenses above were designed to shape the phase fronts for particular wavelengths, however, to characterize the lens performance, it is necessary to research their deviating behaviors with incident light different to the design wavelengths. As shown in Figure 5a, the simulated focal length shifts of three metalenses (symbols) fit well with their respective diffractive lenses (lines) with the same geometric parameters accordingly, especially at their design wavelengths. Here the diffractive lens’ focal shift is given by:

(6) $$f(\lambda )=\frac{f(\lambda_{0})\lambda_{0}}{\lambda }$$

where *λ* is the incidence wavelengths, *λ*_0_ and *f*_0_ are the design wavelength and focal length, respectively. As expected, the focusing efficiencies decrease when using a wavelength different to their respective design values for the three metalenses (Figure 5b). Figure 5c,d are the intensities distribution (in false colors corresponding to their respective wavelengths) in the x-z plane at y = 0 of three metalenses at different wavelengths ranging from 400 nm to 700 nm, respectively. The wavelengths of incidence are denoted on each picture. All the intensities are normalized to that of their design wavelength. It is intuitive that when moving away from the design wavelength, the focal spot sizes including widths and depths become larger, which indirectly proves the decrease of the focusing efficiency away from the default wavelength. Besides, the defocusing effect is more obvious at metalens designed for *λ*_0_ = 633 nm. We characterized the performance of this diffractive metalens in terms of its focal spot profiles (Figure 5f). They were simulated at the practical focal plane corresponding to illumination wavelengths from 450 nm to 700 nm, respectively (white dashed line in Figure 5e). Here we need to note that although the focal spots seem like larger with the increase of incidence wavelengths, their according FWHM deviations are slight and within 10% (60 nm) comparing theoretical values of *λ*_0_/2*NA* (Figure 5f,g), which can be calculated by the following,

(7) $$FWHM_{real}=\frac{\lambda }{2NA(\lambda )}=\sqrt{\frac{{\left(\lambda_{0}f_{0}\right)}^{2}}{D^{2}}+\frac{\lambda^{2}}{4}}$$

where *λ* is the incidence wavelengths and *D* is the diameter of the metalens, $NA(\lambda )=D/(2\sqrt{f{\left(\lambda \right)}^{2}+D^{2}/4})$. The FWHM deviation is defined as,

(8) $$\kappa =\frac{FWHM_{real}-FWHM_{theory}}{FWHM_{theory}}\times 100\%$$

Similarly deviations from theoretical FWHMs of metalenses designed for $\lambda_{0}$ = 453 and 532 nm were shown in Figure 5g, about −5 to 10 and −8 to 8 deviations were obtained, respectively, in the visible spectrum.

### 4.2. Polarization-Insensitive Properties

**Figure 6:**
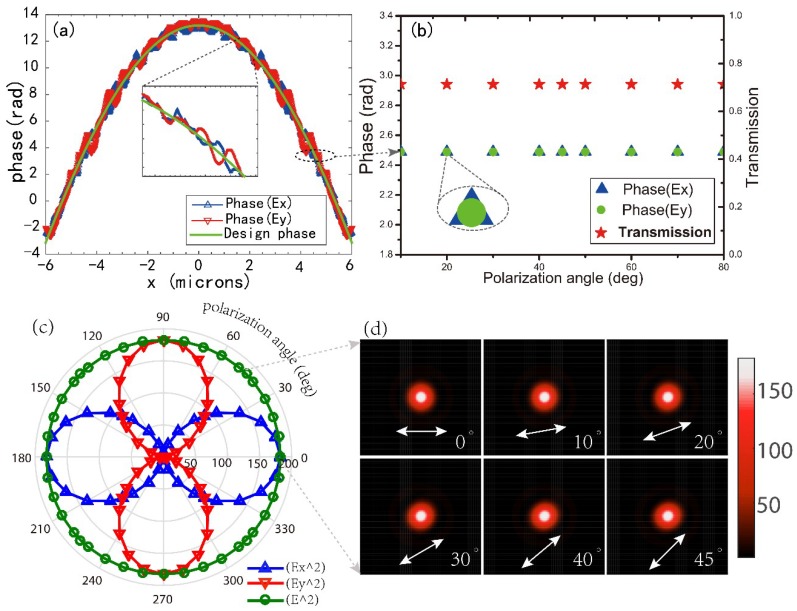
(**a**) The simulated phase profile of x-component (blue curve) and y-component (red curve) and the design phase profile (green curve) according to Equation (1) for the antenna array along the x axis under incidence light with a 10° polarization angle. The inset is a portion of the curves at a higher magnification. (**b**) The phase and transmission amplitude variation under different incidence with polarization angle ranging from 0 to 90° for a single nanopillar extracted in (a). The longitude direction represents the incident polarization. (**c**) The PFIs (green curve) and its x-components (blue curve) and y-components (red curve) contributed by x-polarized light and y-polarized light under different incident lights with polarization angles ranging from 0–360°, respectively. The latitude direction represents the intensity. (**d**) Simulated focal spots of the metalens (NA = 0.51) at its designed wavelength of 633 nm for different linearly polarized inputs ranging from 0 to 45°. The polarization angle is noted on each Figure.

We know that the intensity of linearly polarized incident light can be regarded as the sum of the intensities of its two components: $I={\left|E_{x}\right|}^{2}+{\left|E_{y}\right|}^{2}$. Actually, these two components can independently and simultaneously contribute to the PFI because they can share the same wavefront, the total PFI can be expressed as:

(9) $$\begin{array}{l} PFI=PT_{\lambda }S/\lambda^{2}f^{2}={\int_{S}(\left|E_{x}\right|}^{2}+{\left|E_{y}\right|}^{2})dST_{\lambda }S/\lambda^{2}f^{2}\\ ={\int_{S}\left|E_{x}\right|}^{2}dST_{\lambda }S/\lambda^{2}f^{2}+{\int_{S}\left|E_{y}\right|}^{2}dST_{\lambda }S/\lambda^{2}f^{2}\\ =PFI_{x}+PFI_{y} \end{array}$$

which can be explained by Figure 6a,b: Figure 6a shows the simulated phase profiles of the x-component (blue curve) and y-component (red curve) as well as the design phase profile (green curve) according to Equation (1) for the antenna array along the x axis under the incidence light with 10° polarization angle. We also extract a single nanopillar in Figure 6a and research its transmitted phase and amplitude variation under different incidences with polarization angle ranging from 0 to 90°, as shown in Figure 6b. It is notable that the x-component and y-component transmitted phases and amplitudes of each square nanopillar are consistent with the design values at different polarization angle of incidence, which makes the proposed metalens share the same wavefront for different polarized lights, or in other words, polarization-insensitive to incident light. We can further prove it using the simulated results shown in Figure 6c, although the x-component (blue curve) and y-component (red curve) PFI vary with the different polarization angle of incident light, the total PFI = PFI_x_ + PFI_y_ (yellow curve) remains identical and their focal spots are unchanged (Figure 6d). As a circularly polarized beam can be regarded as a superposition of two linearly polarized beams, whose amplitudes are identical and polarization directions are perpendicular and have phase differences of π/2, the total focusing result can still be viewed as the superposition of two components (see Figure S4), thus the proposed metalenses are also insensitive to circularly polarized inputs.

### 4.3. Comparision of Previously Reported Metalenses and Our Work

As shown in Table 1, we have made some comparisons of the metalenses’ performance between other works and ours by summarizing several representative metalenses that are based on different phase modulation mechanisms and materials at different wavelengths. For first two metalenses based on the phase mechanism of resonance tuning, “V” shaped antennas for example, have been introduced to induce cross components and phase shift by tuning the shape and orientation of each antenna for linearly polarized incidence. This type of metalenses is very thin, however, due to the low conversion efficiency of these applications, a copolarized component still exists in the outgoing field (at best 25% of the total energy can be utilized), and thus these type metalenses suffer from low efficiency. For P-B metalenses, as described in the introduction, they also rely deeply on the cross-polarization of the incident circular polarized light, but their conversion efficiency of polarizations are normally higher than that of metalenses based on resonance tuning. Here we show one of the best P-B metalenses of a past paper [23], in which about 95% of the polarization conversion efficiency promises the realization of focusing efficiency as high as 86%. Therefore, realizing high polarization conversion efficiency is critical to obtain high performance for these type metalenses. For waveguide-type metalenses, they can almost fully utilize the power of incident light, except for the common losses to focus, which enable the metalenses highly efficient at initial design. As shown in a past paper [29], metalenses based on the circular waveguide mechanism (cylindrical nanopillar) and a TiO_2_ material can realize as high as 90% efficiency at visible wavelength. In this work, our SSW-based metalenses can realize polarization-insensitive focusing with efficiency up to 83.4%. Actually, for achieving high focusing efficiency, it is essential to avoid platforms whose efficiencies are limited by absorption losses or fundamental physical limits.

## 5. Conclusions

In conclusion, we have introduced highly efficient metasurfaces in the form of metalenses in the visible range based on SSW theory and a c-TiO_2_ material. Metalenses designed at wavelengths of 453 nm, 532 nm, and 633 nm with numerical apertures (NA) of 0.51 have the capacity of focusing incident light to very symmetric diffraction-limited spots with corresponding efficiencies of 83%, 73.5%, and 78%, respectively. Unlike previous authors who only provided qualitative methods of analysis, we quantitatively analyzed the PFIs and focal spot sizes in the visible spectrum, and the results agree well with theoretical calculations, the simulated focal spot sizes are only about 20 nm lager than the diffraction-limited values (Rayleigh criterion) at different design wavelengths. Compared to the P-B metalenses, our metalenses are polarization-insensitive to incident light, no matter the linear polarization from any state (0–360°) and circular polarization with different chirality. Such an approach can be applied to the design of other metasurface devices like axicons, blazed gratings, and vortex plates and so on that pave the way for the construction of integrated photonic systems.

## Figures and Tables

**Table 1:** Comparison of previously reported metalenses and our work.

Reference	Phase Mechanism	Materials	λ(nm)	Polarization	Thickness(nm)	Efficiency
Aieta et al. [5]	Resonance tuning	Au	1550	Linear (cross *)	60	1%
Ni et al. [40]	Resonance tuning	Au	676	Linear (cross *)	30	10%
Mo et al. [23]	P-B	a-TiO_2_	405, 532, 660	Circular (cross *)	600	86%, 73%, 66%
Shu et al. [21]	P-B	GaN	530	Circular (cross *)	800	67%
This work	Slab Waveguide	c-TiO_2_	453, 532, 633	Insensitive	488	83.4%, 73.5%, 78%
Mo et al. [29]	Circular Waveguide	a-TiO_2_	405, 532, 660	Insensitive	400, 600	30%, 70%, 90%

* Cross: focused light is cross-polarized compared with the incident light.

## Supplementary Materials

The following are available online at http://www.mdpi.com/2079-4991/8/9/699/s1.
