# Supplementary material for: Metalenses Based on Symmetric Slab Waveguide and c-TiO2: Efficient Polarization-Insensitive Focusing at Visible Wavelengths
Source: Nanomaterials (Basel). 2018 Sep 7;8(9):699. doi: 10.3390/nano8090699 (PMC6165503; doi:10.3390/nano8090699)
Supplement: Supplementary file 1 [file nanomaterials-08-00699-s001.pdf]

Supplimetary materials for

Metalenses Based on Symmetric Slab Waveguide and c-TiO_2: Efficient Polarization-Insensitive Focusing at Visible Wavelengths

Yaoyao Liang [1,2], Zhongchao Wei [1,2,*], Jianping Guo [1,2], Faqiang Wang [1,2], Hongyun Meng [1,2], Hongzhan Liu [1,2]

[1] Guangzhou Key Laboratory for Special Fiber Photonic Devices, South China Normal University, Guangzhou 510006, China;

[2] Guangdong Provincial Key Laboratory of Nanophotonic Functional Materials and Devices, School of Information and Optoelectronic Science and Engineering, South China Normal University, Guangzhou 510006, China

* Correspondence: wzc@scnu.edu.cn

1. Comparison of wavefronts manipulated with different accuracies.

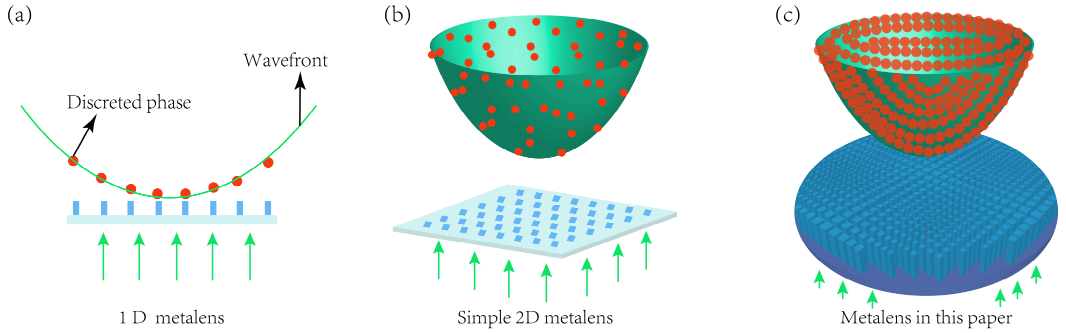

Figure S1 (a) 1D metalenses with several antennas in 1D period. (b) 2D metalenses with dozens of antennas in whole. (c) The metalens proposed in this paper. The red points represent the discrete phase contributed by the antennas, the green curve or surface represent the expected wavefront.

2. Optimization of sampling space at wavelengths of 453, 532, 633nm.

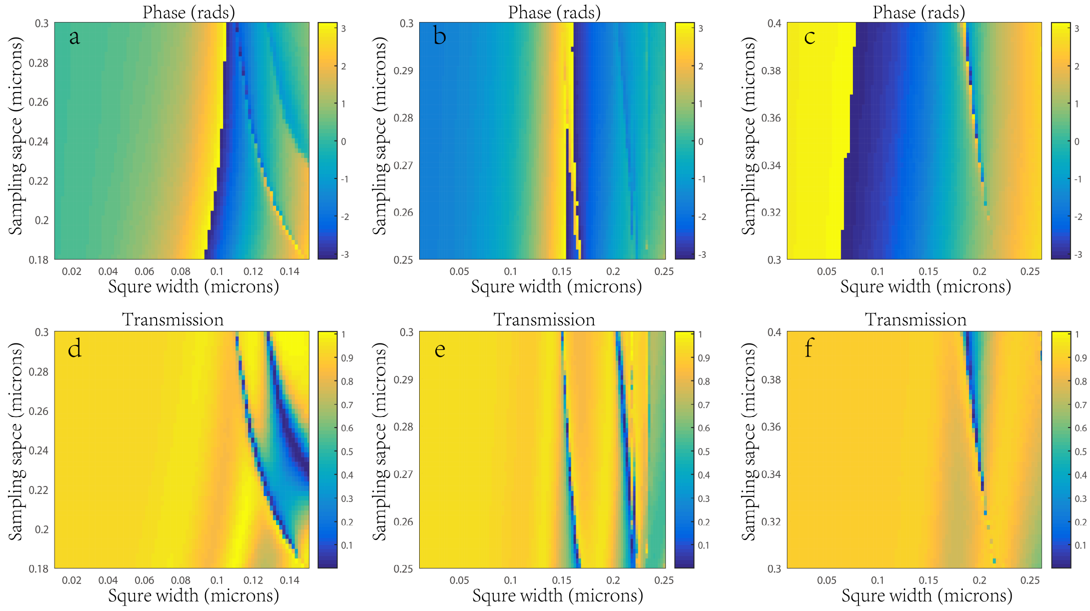

Figure S2 (a-c) are the phase maps $\varphi_{U,w}$ as a function of square widths w and sampling spaces P of nanopillars at the wavelength of 453nm, 532nm, and 633nm, respectively. (d-f) According transmission maps $T_{U,w}$ in (a-c) respectively.

3. Evaluation of the focusing properties.

Here we present a consistent criterion for characterizing the focusing performance of a lens with any combination of geometric parameters. An ideal lens with a focal length f and radius a will produce an intensity profile given by the Airy disk:

$$I(\theta) = I_0 (2\frac{J_1(ka\sin\theta)}{ka\sin\theta})^2 \quad (1)$$

Where $I_0 = PT_\lambda S / \lambda^2 f^2$ is the peak focusing intensity (PFI), J_1 is the first order Bessel function of the first kind, k is the free space wave vector of the incident light, a is the radius of the lens, and θ is the angular position as shown in Figure S3 (a).

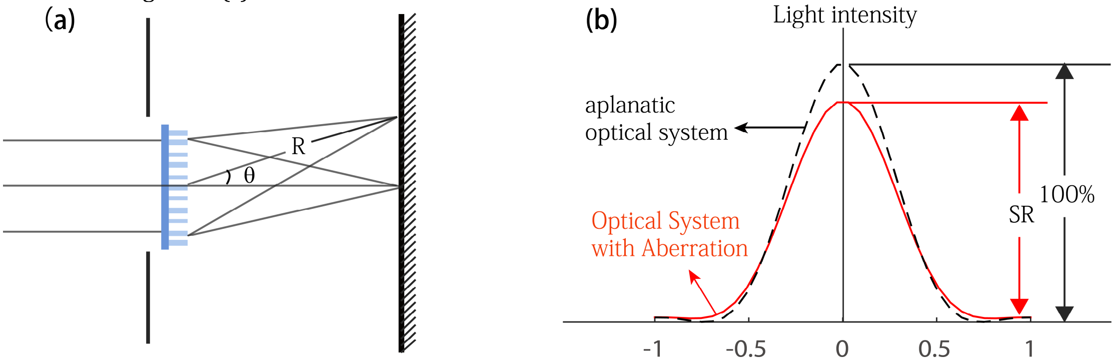

Figure S3 (a) The schematic of diffraction from an aperture with a metalens. θ is the angle of observation, R is the distance from the aperture (b) The demonstration of strehl ratio: here the optical system with aberration and aplanatic optical system can be regarded as the simulated and the ideal intensity profile of the focal spots, respectively. The strehl ratio is ratio is the ratio of simulated PFI to ideal PFI.

To characterize the focusing performance, the simulated intensities of the vertical cuts of focal spots for three metalenses are normalized to those of ideal Airy functions with the same area under the curve respectively as shown in figure 3(g-i) in the manuscript. By comparing the simulated and ideal PFIs, we can obtain the strehl ratio (SR) for three metalenses respectively. The width of the airy disk is used to define the theoretical physical limit to the size of the focal spot achievable by a perfect circular lens, and is defined by the first zero of the airy disk. Once two airy disks become any closer than half their widths, they are also no longer resolvable (Rayleigh criterion). This limit depends both on the geometry of the lens and the wavelength of light, and is given by:

$$\Delta x = 1.22\lambda N \quad (2)$$

Where Δx is the radius of the diffraction-limited spot, λ is the wavelength of interest, and N is the f-number of the lens and is defined as the ratio of f / D.

4. Polarzation-insensitive properties to circularly polarized light

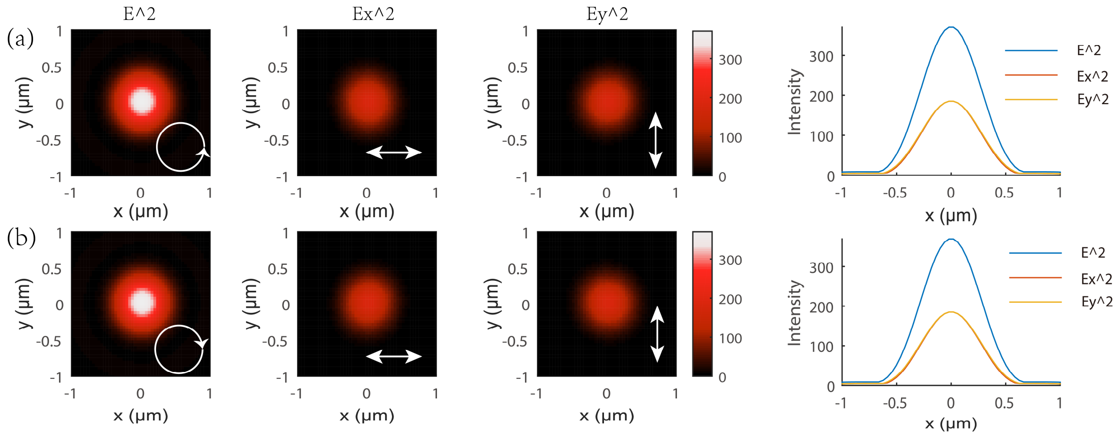

Figure S4 (a,b) the total focusing intensities distributions (E^2) and its x-components (Ex^2) and y-components (Ey^2) distributions in the focal plane as well as the vertical cuts of focal spots under the right and left circularly polarized light, respectively.
